# Tendon transfer in foot drop: a systematic review

**DOI:** 10.1007/s00402-021-04162-x

**Published:** 2021-09-15

**Authors:** Stella Stevoska, Lorenz Pisecky, Christian Stadler, Manuel Gahleitner, Antonio Klasan, Matthias C. Klotz

**Affiliations:** 1 Department for Orthopaedics and Traumatology, Kepler University Hospital GmbH, Johannes Kepler University Linz, Krankenhausstrasse 9, 4020 Linz and Altenberger Strasse 69, 4040 Linz, Austria; 2 Marienkrankenhaus Soest, Orthopedics and Trauma Surgery, Widumgasse 5, 59494 Soest, Germany

**Keywords:** Tibialis posterior tendon, Interosseus membrane, Circumtibial, Fixation technique, Outcome measures

## Abstract

**Introduction:**

Foot drop is a disorder that impairs walking and leads to tripping and falling. Tendon transfer (e.g., tibialis posterior tendon) is a typical secondary procedure in foot drop treatment. The purpose of this systematic review was to identify the most common tendon transfer techniques for treating foot drop and to analyze the reported functional outcomes. Furthermore, it was of interest if the type of surgical technique affects the functional outcome.

**Methods:**

A PubMed and MEDLINE literature search was performed according to PRISMA guidelines. The search terms used were (“tendon transfer” OR “tendon transposition”) AND (“foot drop” OR “peroneal neuropathies”). Any study published before January 2020 was considered for inclusion. No case reports or reviews were included. Common outcome measures (Stanmore score, AOFAS, FAAM, AFO use, patient satisfaction and active ankle dorsiflexion) were evaluated. The quality of the included studies was assessed using the Coleman Methodology Score.

**Results:**

Of the 125 reviewed publications, 37 met the inclusion criteria. 42 cohorts were analyzed. The frequently reported tendon transfer technique was the tibialis posterior tendon transfer through the interosseus membrane. The most used fixation technique was tendon on tendon fixation; however in recent years, a tendon to bone fixation has gained popularity. There was an increase in Stanmore scores and AOFAS postoperatively and a decrease of AFO use postoperatively observed.

**Conclusions:**

Due to various outcome measures and lack of preoperative assessment in the included studies, a meta-analysis of the pooled results was not possible. Nevertheless, the findings of this study show that tendon transfer increases mobility and self-independency leading to patient satisfaction. The choice of the surgical technique does not affect the outcome. A prospective collection of patient data and standardized outcome measures will be important to further analyze the efficacy of tendon transfer techniques.

## Introduction

A foot drop is defined as a loss of active dorsiflexion at the tibiotalar joint, which is compensated by hyperflexion in the hip and knee joints. Furthermore, internal rotation of the foot in the transverse plane is also a usual compensatory mechanism [1, 2]. This so called steppage gait impairs mobility and quality of life due to an increased risk of tripping and falling [1, 3]. Several reasons like injuries, neuromuscular disorders or anatomical variations may lead to foot drop [1]. The most common cause is a (common) peroneal neuropathy at the neck of the fibula [1, 4]. The treatment depends on the cause and severity of foot drop. For patients with a severe foot drop of any cause, an ankle–foot orthosis (AFO) is a helpful device that enables them to improve gait and helps to prevent falls. Primary nerve procedures such as nerve decompression, nerve repair, and reconstruction are treatment options if the cause is known and it could be treated [1, 2]. However, for patients with foot drop that lasts for more than 1 year with little chance of motor function improvement, secondary procedures like tendon transfers can be considered [1, 4].

In 1933, Ober [5] first described tibialis posterior tendon transfer routed around the tibia and subcutaneously around the ankle to the dorsum of the foot (circumtibial route). In 1937, Mayer [6] credited Putti with the description of tibialis posterior tendon transfer through the interosseous membrane to the dorsum of the foot. Carayon et al. [7] in 1967 described a dual transfer—interosseously transferred tibialis posterior tendon sutured to tibialis anterior tendon and the flexor digitorum longus tendon sutured to both the extensor hallucis longus and extensor digitorum longus tendons [7]. Srinivasan et al. [8] in 1968 split the tibialis posterior tendon into ‘two’ tails. The medial tail was inserted into the tendon of extensor hallucis longus and the lateral tail into the tendons of extensor digitorum longus and peroneus tertius [8]. In 1991, the first clinical series of the Bridle procedure (Riordan technique) was reported by McCall et al. [9]. It consists of a tibialis posterior transfer through the interosseous membrane and a concomitant subcutaneous peroneus longus transfer with the tri-tendon double-end-weave anastomosis between tibialis posterior, tibialis anterior and peroneus longus tendon [9]. Rodriquez [10] modified the Bridle procedure by additionally transferring the attachment of the anterior tibial tendon to the second cuneiform bone.

There are several studies reporting the outcome after tendon transfers in foot drop. Nevertheless, the level of evidence of such studies is poor. In addition to that, as above mentioned, several different techniques are available. Therefore, the purpose of this systematic review was to identify most common tendon transfer techniques in case of foot drop and to analyze the functional outcome. Furthermore, it was of interest if the type of tendon transfer technique affects the functional outcome.

## Methods

### Search strategy and criteria

The study was conducted according to PRISMA guidelines [11]. A comprehensive systematic literature search was undertaken to identify relevant efficacy and safety data reported for the tendon transfer for correction of foot drop. The search of the literature was carried out using electronic database PubMed and MEDLINE. All studies published until January 2020, when this study was conducted, were screened for inclusion. The search terms used were (“tendon transfer” OR “tendon transposition”) AND (“foot drop” OR “peroneal neuropathies”) in which all capitalized words represent the Boolean operators used. In addition, all references cited in identified reviews were manually searched for potentially relevant studies and considered for inclusion.

### Inclusion and exclusion criteria

The literature was screened for studies that included subjects (no age limitation), who underwent tendon transfer for correction of foot drop, with or without additional procedures. No studies were excluded based on follow-up time, although most studies followed patients clinically for at least six months.

Identified studies were included if they assessed at least one of the following outcome measures: (1) frequent clinical scores, such as Stanmore score, AOFAS and FAAM; (2) AFO use in daily life activities; (3) patient satisfaction; (4) either active or passive range of ankle motion, using a goniometer. Other outcome measures than those mentioned above were ineligible.

The study had to be published either in the English or German language. There were no restrictions on the year of publication.

Duplicates, abstracts, case reports, conference presentations and publications including cohorts with less than five patients were excluded. Publications with non-existent outcome measures and publication reporting about gastrocnemius muscle transfer technique were also excluded. Additionally, studies without an available full-text article were excluded.

### Assessment of study quality

The methodological quality of the included studies was independently assessed by two authors—junior and senior doctor (i.e., observers) using the same consensus rule. The evaluation system adopted was the Coleman Methodology Score (CMS) [12]. Observers could not find a possibly more appropriate and validated modification with open excess; therefore, the original CMS was adopted after consultation with other authors [12]. Each study was given a Coleman Methodology Score of between 0 and 100 after scoring for 10 criteria.

### Data collection and abstraction

All citations and abstracts identified from the PubMed and MEDLINE literature search, as well as manually searched potentially relevant studies, were screened against the eligibility criteria.

For each publication, two observers—junior and senior doctor—independently assessed records by title and abstract for eligibility for inclusion, based on four criteria: population, intervention, outcome measures and study design. Any disagreement on inclusion of publications was discussed between observers until consensus was reached. After initial screening of these abstracts, observers assessed the remaining full-text articles for inclusion in the study. First author, junior doctor, extracted data from each included study. Any discrepancies were discussed between observers until consensus was reached.

For all eligible studies, the following study characteristics were extracted: publication year, author, study design, cohort, number of patients and/or feet in each cohort, follow-up time, operative technique, subgroups (if applicable), additional procedures and all eligible outcome measures. Data were extracted from article texts, tables, and figures.

Potential differences in outcomes, attributable to the transfer and fixation technique selection, were not further evaluated, if different transfer or fixation techniques were used in the same cohort Table 1.

**Table 1:** Study characteristics—first author, year of publication, study type and follow-up time

Study no	Author	Year of publication	Study type	Follow-up time (months)		
				Mean	Minimal	Maximal
1	Sturbois-Nachef et al. [38]	2019	Retrospective	66	12	108
2	Lingaiah et al. [25]	2018	Prospective	NA	24	NA
3	Vieira et al. [24]	2018	Retrospective	NA	5	NA
4	Cho et al. [4]	2017	Retrospective	65.6	36	NA
5	Werner et al. [19]	2017	Retrospective with prospective follow-up	32	25	39
6	Movahedi Yeganeh et al. [39]	2016	Retrospective	NA	12	50
7	Flynn et al. [40]	2015	Retrospective	61	31	144
8	Johnson et al. [35]	2015	Retrospective	22.8	12	NA
9	Molund et al. [20]	2014	Retrospective	56	8	204
10	Ho et al. [21]	2014	Retrospective	21	3	48
11	Dreher et al. [41]	2014	Prospective	28.8	NA	NA
12	Yeganeh et al. [22]	2013	Retrospective	6	NA	NA
13	Das et al. [33]	2013	Retrospective with prospective follow-up	23.6	NA	NA
14	Aydin et al. [23]	2013	Retrospective	32.54	24	55
15	Mehling et al. [42]	2012	Retrospective	64	6	138
16	Reis et al. [43]	2012	Retrospective	27.6	12	60
17	Cohen et al. [14]	2012	Retrospective	NA	NA	NA
18	Elsner et al. [44]	2011	Retrospective	48	46	70
19	Steinau et al. [45]	2011	Retrospective	78	24	156
20	Kremer et al. [36]	2011	Retrospective	40.8	10	90
21	Özkan et al. [46]	2009	Retrospective	101	28	132
22	Fuhrmann et al. [47]	2009	Retrospective	12	NA	NA
23	Shah et al. [48]	2009	Prospective	48	6	85
24	Vigasio et al. [49]	2008	Retrospective	65	24	144
25	Wagenaar et al. [15]	2007	Retrospective	44.4	9	81
26	Ishida et al. [13]	2007	Retrospective	29.1	10	48
27	Yeap et al. [31]	2001	Retrospective	64.6	7,5	300
28	Yeap et al. [34]	2001	Retrospective	90	24	300
29	Hove et al. [37]	1998	Retrospective	24	12	60
30	Bari et al. [50]	1996	Retrospective	NA	6	24
31	Soares et al. [51]	1996	Retrospective	31	6	85
32	Prahinski et al. [52]	1996	Retrospective	61	14	118
33	Rodriguez et al.[10]	1992	Retrospective	80	6	156
34	Richard et al. [53]	1989	Retrospective	NA	NA	NA
35	Pinzur et al. [54]	1988	Retrospective	35	24	56
36	Hall et al. [55]	1977	Retrospective	NA	3	NA
37	Srinivasan et al. [8]	1968	Retrospective	12	6	39

*No *number, *NA *not available

If only a number of patients were available, it was considered to be the same as the number of operated feet (Table 2). If the number of patients who used an ankle–foot orthosis was not specifically defined preoperatively, it was assumed that all patients in the cohort used the orthosis in daily life activities preoperatively.

**Table 2:** Overview over cohorts—number of patients/feet and surgical technique characteristics

Cohort no	Author	Patients	Feet	TPT		Fixation		
				CT route	IO route	Tendon	Bone	Tendon and bone
1	Sturbois-Nachef et al. [38]	13	NA		IO	Tendon		
2	Lingaiah et al. [25]	30	NA		IO		Bone	
3	Vieira et al. [24]	7	NA		IO		Bone	
4	Cho et al. [4]	17	NA		IO		Bone	
5	Werner [19]	5	NA		IO		Bone	
6	Movahedi Yeganeh et al. [39]	15	NA		IO			Tendon and bone
7	Flynn et al. [40]	8	9		IO	Tendon		
8	Johnson et al. [35]	19	NA		IO			Tendon and bone
9	Molund et al. [20]	12	NA		IO		Bone	
10	Ho et al. [21]	12	NA		IO		Bone	
11	Dreher et al. [41]	14	23		IO	Tendon		
12	Yeganeh et al. [22]	15	NA		IO		Bone	
13A	Das et al. [33]	NA	162	CT		Tendon		
13B	Das et al. [33]	NA	219		IO	Tendon		
14	Aydin et al. [23]	24	NA	CT			Bone	
15	Mehling et al. [42]	14	NA		IO	Tendon		
16	Reis et al. [43]	13	NA	CT		Tendon		
17	Cohen et al. [14]	19	NA			Tendon	Bone	
18	Elsner et al. [44]	19	NA		IO		Bone	
19A	Steinau et al. [45]	31	NA		IO	Tendon		
19B	Steinau et al. [45]	20	NA		IO	Tendon		
20	Kremer et al. [36]	13	NA		IO	Tendon		
21	Özkan et al. [46]	16	NA	CT		Tendon		
22	Fuhrmann et al. [47]	6	NA		IO		Bone	
23	Shah et al. [48]	69	120		IO	Tendon		
24	Vigasio et al. [49]	16	NA		IO	Tendon		
25	Wagenaar et al. [15]	12	13		IO	Tendon		
26A	Ishida et al. [13]*	33*	NA	CT	IO	Tendon		
26B	Ishida et al. [13]	19	NA	CT		Tendon		
26C	Ishida et al. [13]	14	NA		IO	Tendon		
27	Yeap et al. [31]	18	NA		IO	Tendon	Bone	
28	Yeap et al. [34]	12	NA		IO	Tendon		
29	Hove et al. [37]	17	20	CT	IO	Tendon	Bone	
30	Bari et al. [50]	20	NA	CT		Tendon		
31A	Soares et al. [51]	NA	26	CT		Tendon		
31B	Soares et al. [51]	NA	43		IO	Tendon		
32	Prahinski et al. [52]	10	NA		IO	Tendon		
33	Rodriguez et al. [10]	10	11		IO			Tendon and bone
34	Richard et al. [53]	29	39		IO	Tendon		
35	Pinzur et al. [54]	9	NA		IO		Bone	
36	Hall et al. [55]	54	65	CT	IO	Tendon		
37	Srinivasan et al. [8]	33	39	CT		Tendon		
Total number (%)		1227		11 (26%)	33 79%)	28 (67%)	14 (33%)	3 (7%)

*No*. number, *NA* not available, *TPT *tibial posterior tendon transfer, *CT *circumtibial, *IO *interosseous membrane

*Cohort with total number of patients

## Results

### Search results

**Fig. 1:**
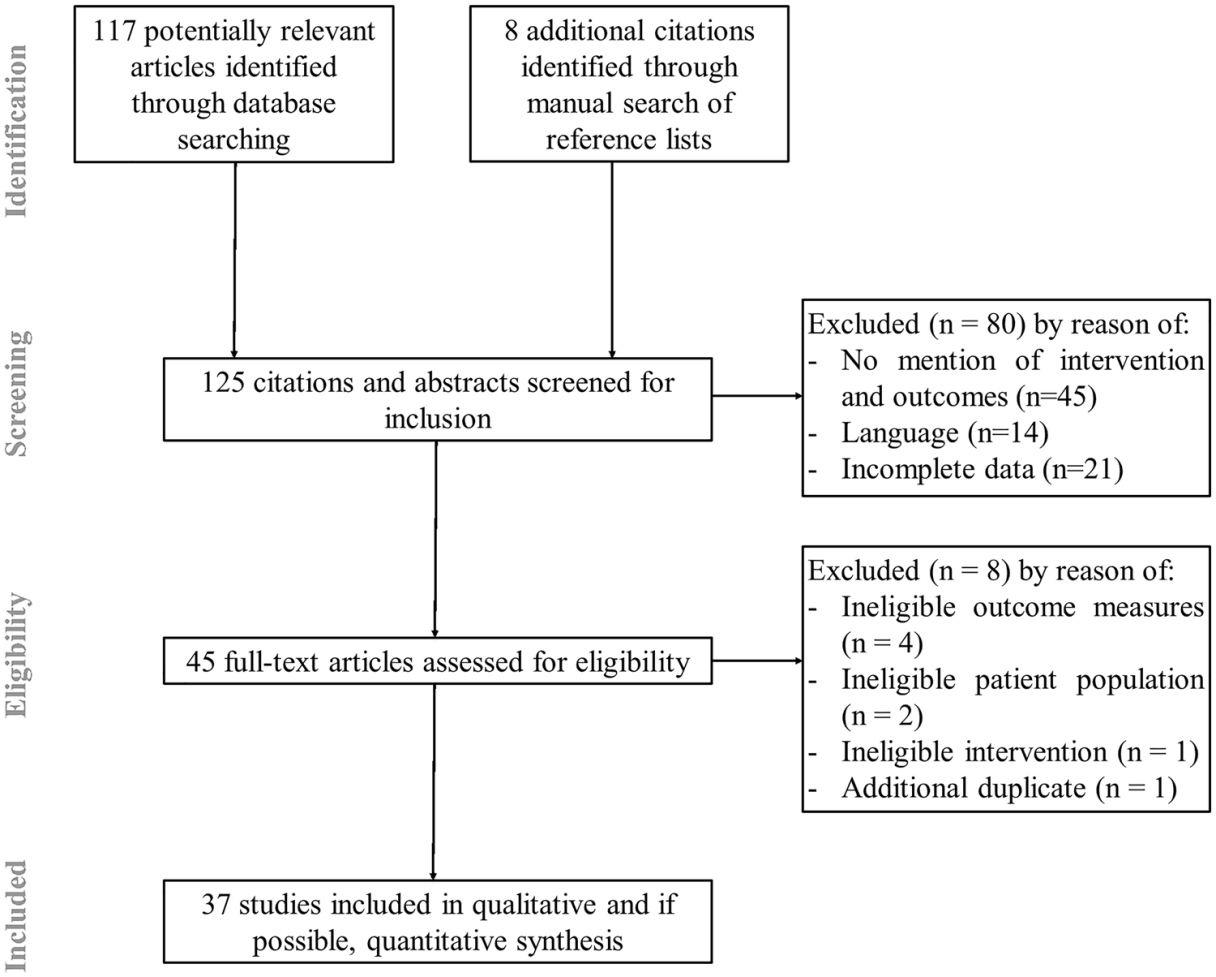
Preferred Reporting Items for Systematic Reviews and Meta-Analyses (PRISMA) flowchart showing the selection process

A total of 117 potentially relevant articles were identified from the electronic database search after duplicates were removed (Fig. 1). An additional eight citations were considered for inclusion through the scanning of reference lists of published reviews or studies of interest, leaving a total of 125 potentially relevant citations for screening. After removal of duplicates, and studies that did not fit the eligibility criteria, 37 studies were included for review (Fig. 1).

The average value of CMS was 67.2 ± 9.7. Studies that did not score well included retrospective studies with fewer patients, shorter follow-up and poor reporting of patient recruitment. The majority of studies lost points from their CMS scores as a result of the study investigators being the surgeons who performed the operations.

### Cohort characteristics

The characteristics of the included studies are summarized in Tables 1 and 2. A total of 1227 feet (mean age, 35 years; range, 12–55; SD, 8) out of 37 publications (42 cohorts) were included. The publication years ranged from 1968 to 2019 (32 retrospective, 3 prospective and 2 retrospective studies with prospective follow-up), with the range of mean follow-up time between 6 and 101 months (data available in 81% of included studies, 30/37). Four publications included multiple cohorts—two cohorts: 3; three cohorts: 1 (Tables 1 and 2). In one study [13], three cohorts were assessed: cohort with a total number of patients and two subgroups—cohort with tibialis posterior transfer through the interosseous membrane (IO) and cohort with tibialis posterior transfer through the circumtibial route (CT). The cohort with a total number of patients was evaluated with different outcome measures as subgroups. Hence, all three cohorts were included (Table 2). Therefore, out of the 37 publications, 42 cohorts were analyzed.

Indication for a tibialis posterior tendon transfer was mostly a foot drop caused by common peroneal nerve palsy (33/37 studies, 89%), either due to traumatic injury (20/37 studies, 54%) or due to infective neuropathy caused by leprosy (11/37 studies, 30%). In 3 of 37 studies (8%) an exact reason for CPN palsy was not clearly reported. Central neurological or neuromuscular disorders like Charcot–Marie–Tooth disease were reported as a reason for a foot drop occurrence in 7 of 37 studies (19%). Several studies included patients with various disorders in the same cohort. The treatment approach was not adjusted according to foot drop etiology.

### Operative technique characteristics

The transfer of tibialis posterior tendon through the interosseus membrane (IO) was used in 33 of 42 cohorts (79%) and the tibialis posterior transfer through the circumtibial route (CT) in 11 of 42 cohorts (26%). Three of the above-mentioned cohorts used both types of transfers in the same cohort. Only one cohort, Cohen J. C. et al. [14], reported a transfer of peroneus longus tendon (Table 2).

**Table 3:** An overview of the results (in points) of the most commonly used scoring systems: Stanmore score, AOFAS and FAAM (including Activities of Daily Living Subscale and Sports Subscale)

	*n*	Mean preoperative value ± SD	*n*	Mean postoperative value ± SD	n	Mean difference* ± SD
Stanmore score	4	24 ± 13	11	78 ± 8	4	56 ± 14
AOFAS	3	53 ± 10	7	76 ± 11	3	26 ± 8
FAAM total	1	46	3	73 ± 12	NA	NA
FAAM ADL	1	59	3	86 ± 11	NA	NA
FAMM sports	1	33	3	60 ± 14	NA	NA

Table includes preoperative and postoperative values. For the studies where both values were available also the difference was computed

^*^The mean difference of the mean preoperative and the mean postoperative values for the studies where both values were available. *N *the number of studies evaluated by scoring, *SD *standard deviation, *AOFAS *The American Orthopaedic Foot and Ankle Society Ankle-Hindfoot Scale, *FAAM *Foot and Ankle Ability Measures, *ADL *Activities of Daily Living Subscale, *NA *not available

Three types of fixations were used—tendon to tendon fixation in 28 of 42 cohorts (67%), tendon to bone in 14 of 42 cohorts (33%) and the combined fixation technique in 3 of 42 cohorts (7%). In 3 of 42 cohorts (7%) a tendon to tendon and tendon to bone fixation were used in the same cohort. Eight of 11 bone fixations were reported in studies published after 2012 and were in recent years more common than tendon fixations (Table 2).

If the equines component of the foot was present due to a long term and conservatively probably not optimally treated foot drop, a lengthening of the shortened Achilles tendon was additionally required to achieve the desired function. An additional Achilles’ tendon lengthening (i.e., ATL) was performed in 737 of 1227 feet (60%).

### Outcome characteristics

All extracted data were reviewed and analyzed by our University Department of Statistics. Performance of the appropriate statistical tests reporting *p* value was not possible. Thus, only descriptive statistics of the obtained data were reported.

### Scores

Functional outcome was commonly reported using Stanmore score (*n* = 11), AOFAS (*n* = 7) and FAAM (*n* = 3). However, preoperative values were available only in eight studies (Table 3).

There was an increase in Stanmore score and AOFAS postoperatively (Tables 3, 4). The postoperative values of Stanmore score for subgroups defined by the transfer and the fixation techniques were similar (Table 4).

**Table 4:** Values of different outcome measures according to the transfer route and according to the fixation technique, including preoperative values, if available

			*n*	Mean preoperative value ± SD*	*n*	Mean postoperative value ± SD
Stanmore score (points)	Total		4	24 ± 13	11	78 ± 8
	Transfer route	CT route	2	30	3	80 ± 5
		IO route	2	18	8	77 ± 9
	Fixation technique	Tendon	2	31	5	74 ± 16
		Bone	2	17	4	81 ± 9
		Tendon and bone	NA	NA	1	78
AFO use (%)	Total		22	93 ± 19	22	8 ± 10
	Transfer route	CT route	3	72 ± 49	3	7 ± 8
		IO route	19	97 ± 8	19	8 ± 11
	Fixation technique	Tendon	10	86 ± 27	10	13 ± 11
		Bone	8	100	8	5 ± 10
		Tendon and bone	2	100	2	0
Patient satisfactio*n* (%)	Total				13	94 ± 9
	Transfer route	CT route			3	96 ± 7
		IO route			10	93 ± 10
	Fixation technique	Tendon			5	92 ± 13
		Bone			5	98 ± 4
		Tendon and bone			1	100

*N *the number of cohorts evaluated, *SD *standard deviation, *CT *circumtibial, *IO* interosseus membrane, *AFO *ankle foot orthosis, *NA *not available

^*^Standard deviation was computed only if a minimum of three values were available

### Ankle–foot orthosis (AFO) use in daily life activities

Preoperative and postoperative AFO use were described in 22 of 42 cohorts (52%). In the analyzed 22 cohorts, 298 of 320 patients used AFO preoperatively (93%). Majority of patients, 91% (270/298), who used AFO in daily life activities preoperatively, did not use it postoperatively. However, 3% (8/298) patients still needed AFO occasionally for some activities like sports (Table 4). The percentage of patients with AFO use for subgroups defined by the transfer and the fixation techniques showed similar results between postoperative values (Table 4).

### Patient satisfaction

Patient satisfaction was reported in 13 of 42 cohorts (31%). In total, 94% of patients (205/219) were satisfied with the outcome of the operation (Table 4). For 7 of 13 cohorts (127 patients), the level of satisfaction (very satisfied, satisfied, satisfied with reservations, dissatisfied) was additionally reported. Patients were mostly either very satisfied (55/127, 43%) or satisfied (47/127, 37%). Patient satisfaction defined by the transfer and the fixation techniques showed similar results postoperatively.

### Motion of ankle joint

In 29 cohorts (69%), the postoperative ankle range of motion was reported. Active ankle dorsiflexion was reported in 26, and active range of motion in 23 of 29 cohorts (Table 5). Measurements were performed with either the knee extended or flexed. In many cases, the method of measurement was not reported in detail.

**Table 5:** An overview of preoperative and postoperative values of ankle active dorsiflexion (DF) and ankle active range of motion (ROM) divided in cohorts according to the position of the knee at the time of measurement

	*n*	Mean preoperative value in degrees*	*n*	Mean postoperative value in degrees ± SD
Active DF—extension	2	– 33.2	9	4.5 ± 6.0
Active DF—flexion	2	0.0	5	7.5 ± 3.8
Active DF—unknown	NA	NA	13	5.3 ± 5.9
Active ROM—extension	NA	NA	8	32.7 ± 11.3
Active ROM—flexion	2	11.5	4	18.0 ± 4.7
Active ROM—unknown	NA	NA	11	27.6 ± 13.9

*n *the number of cohorts evaluated, *SD* standard deviation, *DF* dorsiflexion, *ROM* range of motion, *NA* not available

Additionally—for the data available—the values of active dorsiflexion for subgroups defined by the transfer and the fixation techniques are provided in Table 6.

**Table 6:** Comparison of active dorsiflexion measured in different knee positions according to the transfer and according to the fixation technique, including preoperative values, if available

		*n*	Mean preoperative value in degrees*	*n*	Mean postoperative value in degrees ± SD
CT route	Active DF—extension	1	– 33.8	2	9.3
	Active DF—flexion	NA	NA	3	6.8 ± 3.9
	Active DF—unknown	NA	NA	4	5.8 ± 6.3
IO route	Active DF—extension	1	– 32.5	6	2.0
	Active DF—flexion	NA	NA	3	6.9 ± 4.6
	Active DF—unknown	1	– 30	10	4.7 ± 5.9
Tendon fixation	Active DF—extension	1	– 33.8	6	1.7 ± 5.3
	Active DF—flexion	2	0	5	7.5 ± 3.8
	Active DF—unknown	NA	NA	8	4.7 ± 6.0
Bone fixation	Active DF—extension	1	– 32.5	2	10.4
	Active DF—flexion	NA	NA	NA	NA
	Active DF—unknown	1	– 30	3	5.6 ± 5.1
Tendon and bone fixation	Active DF—unknown	NA	NA	1	14.6

*n* the number of cohorts evaluated, *SD* standard deviation, *CT* circumtibial, *IO* interosseus membrane, *DF* dorsiflexion, *NA* not available

Other commonly observed values were passive dorsiflexion, passive or active plantarflexion and passive range of motion (ROM).

## Discussion

This systematic review summarized and analyzed tendon transfer and fixation techniques in foot drop management as well as revealed and analyzed commonly reported outcome measures. In 2007, Wagenaar et al. [15], shortly summarized techniques and outcomes of the foot drop management in discussion section of their retrospective study. According to the literature search, no other systematic review or a meta-analysis on this topic has been published thus far.

### Operative techniques

Anterior transfer of a tibialis posterior tendon appears to be a preferred choice in operative treatment of a persistent foot drop. Various techniques and modifications of the latter have developed over the years. For a more transparent review of the various techniques a division in subgroups was acquired (Tables 2).

**Fig. 2:**
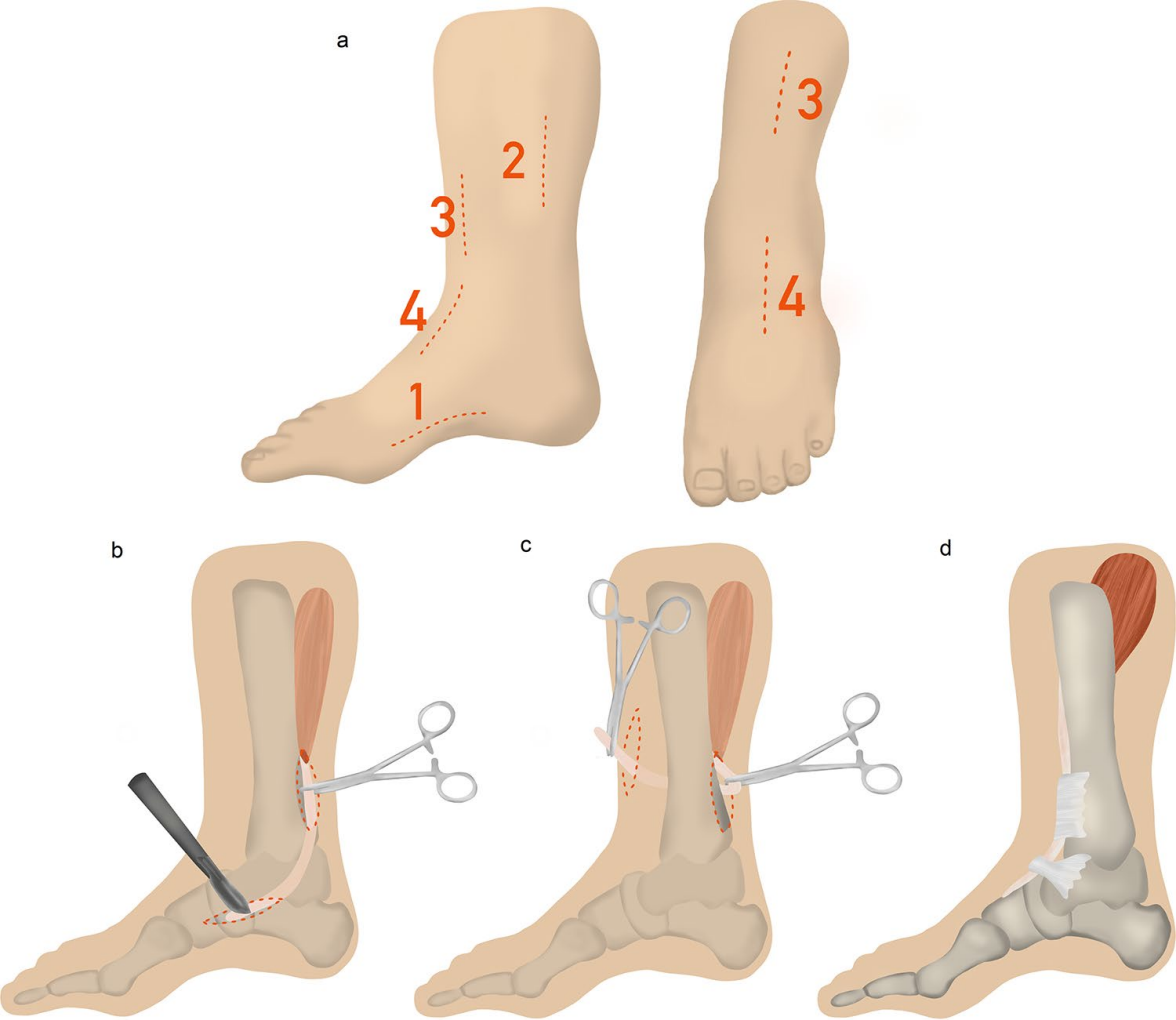
Tibialis posterior tendon transfer through the interosseus membrane and bone fixation. **a** Medial and anterior skin incisions in numerical order; **b** release of navicular insertion (first incision) and exposure of the proximal part of the tibialis posterior tendon (second incision); **c** tibialis posterior tendon passed from posterior to tibia (second incision) through the interosseus membrane into the anterior compartment (third incision); **d** tibialis posterior tendon passed under the retinaculum into the dorsal foot and anchored into the middle cuneiform (fourth incision)

The most commonly reported type of tendon transfer in foot drop management was a tibialis posterior tendon transfer through the interosseus membrane (IO). There are two main variations of posterior tendon transfer through the interosseus membrane (IO) described open and closed transfer [16]. In both variants, the operative procedure can be divided into four steps—disinsertion, relocation, preparation for reinsertion and reinsertion (Fig. 2). The main difference occurs in the second step. In the open procedure described by Watkins et al. 1954 [17], the interosseous membrane is exposed and prepared from the ventral side under view. In the variant described in 1978 by Hsu et al. [18], the interosseous membrane is perforated blindly from the posterior side forward. The authors of this article use the latter variant, as it allows an early mobilization and, if performed correctly, rarely leads to complications (Fig. 2).

The usual type of fixation was tendon to tendon fixation; however in recent years, a tendon to bone fixation has gained popularity [4, 19–25]. With the development of new implants, the attachment of the tendon graft to the bone has been improved. The most recent techniques involve the use of bone anchors or interference screws. Their use minimizes the risk of wound complications or skin ulcerations in comparison to staples or button fixation on the plantar surface. Additionally, compared to tendon fixation or fixation through bone tunnels, the use of a shorter tendon graft is sufficient and less surgical dissection is required [26–30]. The outcomes of the Marsland et al. [27] study find that the strengths of both, interference screw (bone) fixation and Pulvertaft weave (tendon) fixation of the posterior tibial tendon were comparable. However, Pulvertaft weave fixation showed greater variability, indicating it may be a less reliable technique. Screw fixation might therefore be preferable to a tendon fixation as it is less dependent upon surgeon technique.

The points of fixation may vary depending on the muscular imbalance. Preferred points of fixation are either middle/lateral cuneiform or cuboid bone. When patients have a significant weakness of eversion, the posterior tibial tendon should be transferred more to the lateral side of the foot, suturing to either the peroneus brevis or peroneus tertius tendons [27, 30].

### Outcome measures

Numerous outcome measures have been used to evaluate and report the outcome of tendon transfer in the foot drop treatment. Stanmore score, reported from Yeap in 2001 [31], is based on the aims of tibialis posterior tendon transfer. It was the most frequent score in this review (11 cohorts, 26%). The American Orthopaedic Foot and Ankle Society Ankle-Hindfoot Scale [32] (AOFAS) was reported in seven cohorts (17%). AOFAS was designed to assess the outcome of surgery for painful conditions of the foot and ankle [32]. It does not assess tibialis posterior tendon transfers adequately. An active ankle dorsiflexion was the most regularly assessed outcome measure (26 cohorts, 62%). However, the knee position in the measurement was not standardized and not always adequately reported. Ankle–foot orthosis (AFO) use in daily life activities (22 cohorts, 52%) and patient satisfaction (13 cohorts, 31%) were also important outcome measures evaluating mobility, independency and quality of life.

Due to various outcome measures and lack of preoperative assessment in the studies, a clear and high-quality statistical analysis was not possible. Nevertheless, a comparison of Stanmore score, AOFAS and AFO use in daily life activities, before and after surgery was possible. There was a relevant increase in the aforementioned scores and relevant decrease of AFO use. Therefore, it seems that a tendon transfer contributes greatly to patient satisfaction, mobility and quality of life (Tables 3 and 4).

Only one study directly compared the outcome between circumtibial and interosseus route of tibialis posterior tendon transfer [33]. No study was designed to compare the outcome in case of different types of fixation techniques. Therefore, no meta-analysis could be performed on this topic. The choice of the tendon transfer or fixation technique does not appear to affect the outcome (Tables 4 and 6).

This study has a number of limitations. A large number of the analyzed studies (92%) were retrospective with a limited sample size. No randomized controlled clinical trials (RCT) were identified, although RCTs are logistically extremely difficult for such a rare diagnosis. Some of the studies (24%) included in this review were conducted over 20 years ago; however, the reported results seemed consistent with more recent studies included. The diversity of the population was also not taken into account. Additionally, the two publications by the same first author [31, 34], both published in 2001, might have an overlap in the patients of the cohorts. However, there was no overlap of outcome measures. In both cohorts, the transfer procedure is tibialis posterior tendon through the interosseous membrane (IO) (Table 2). Only studies published in English or German were included.

The assumption that all patients used an orthosis before the procedure, if the preoperative documentation was not clear enough, may affect the data on the use of the orthosis. A caution should be taken when interpreting the results. However, the use of the orthosis is a basic conservative measure in the foot drop treatment to prevent contractures and to allow sufficient mobility of the patient. Furthermore, dissatisfaction and insufficient mobility with the orthosis was a common or even decisive indication for surgery in the cohorts analyzed. Therefore, these cohorts were also included [14, 20, 25, 35–37].

Additional procedures, mainly the Achilles tendon lengthening, might impact the outcome results, especially the range of movement. Performing additional procedures in foot surgery at the usual complexity of deformities is often necessary to achieve the desired function. There are no studies that report only tibialis posterior tendon transfer without additional procedures, so the inclusion of these feet was necessary. Although this is not optimal for the analysis of the success of the main procedure itself, the non-performance of additional procedures would limit the foot function, and the interpretation of the results would therefore be limited due to other coexisting deformities.

The outcome measures used in the studies demonstrated a large heterogeneity. Thus, the heterogeneity and poor quality of included studies did not allow a meta-analysis. However, descriptive analyses were conducted to provide more data about the characteristics and the efficacy of tendon transfer in foot drop. In addition, subgroup analyses were performed to overcome the heterogeneity among studies, clarify the review findings and minimize the bias to the lowest possible extent.

## Conclusions

In this systematic review of tendon transfer techniques in foot drop management, a tendon transfer through interosseus membrane and in recent years tendon on bone fixation were the preferred techniques of choice. Due to the heterogeneity and deficiency of data, this report provides a descriptive analysis of the collected data and not a meta-analysis. Although not statistically supported, tendon transfer appears to contribute greatly to patient satisfaction and quality of life. The choice of the tendon transfer or fixation technique does not affect the outcome judging by the value of the common outcome measures. A prospective collection of patient data and standardized outcome measures will be important going forward to analyze the efficacy of tendon transfer further. Moreover, the potential differences in outcomes, attributable to the transfer and fixation technique selection, should be characterized.
